# The Inhibitory Effect of Ginger Extract on Ovarian Cancer Cell Line; Application of Systems Biology

**DOI:** 10.15171/apb.2017.029

**Published:** 2017-06-30

**Authors:** Roghiyeh Pashaei-Asl, Fatima Pashaei-Asl, Parvin Mostafa Gharabaghi, Khodadad Khodadadi, Mansour Ebrahimi, Esmaeil Ebrahimie, Maryam Pashaiasl

**Affiliations:** ^1^Department of Anatomy, Medical School, Iran University of Medical Science, Tehran, Iran.; ^2^Cellular and Molecular Research Center, Iran University of Medical Sciences, Tehran, Iran.; ^3^Molecular Biology Laboratory, Biotechnology Research Center, Tabriz University of Medical Sciences, Tabriz, Iran.; ^4^Women’s Reproductive Health Research Center, Tabriz University of Medical Sciences Tabriz, Iran.; ^5^Genetic Research Theme, Murdoch Children’s Research Institute, Royal Children's Hospital, The University of Melbourne, Melbourne, Australia.; ^6^Department of Biology, University of Qom, Qom, Iran.; ^7^Institute of Biotechnology, Shiraz University, Shiraz, Iran.; ^8^School of Biological Sciences, Faculty of Science and Engineering, Flinders University, Adelaide, Australia.; ^9^School of Information Technology and Mathematical Sciences, Division of Information Technology, Engineering and the Environment, The University of South Australia, Adelaide, Australia.; ^10^School of Animal & Veterinary Science, The University of Adelaide, Australia.; ^11^Drug Applied Research Center, Tabriz University of Medical Sciences, Tabriz, Iran.; ^12^Department of Anatomical Sciences, Faculty of Medicine, Tabriz University of Medical Sciences, Iran.

**Keywords:** Ovarian cancer, Ginger extract, Anticancer, P53, Bcl-2, Systems biology analysis, Meta-analysis

## Abstract

***Purpose:*** Ginger is a natural compound with anti-cancer properties. The effects of ginger and its mechanism on ovarian cancer and its cell line model, SKOV-3, are unclear. In this study, we have evaluated the effect of ginger extract on SKOV-3.

***Methods:*** SKOV-3 cells were incubated with ginger extract for 24, 48 and 72 hours. Cell toxicity assay was performed. Different data mining algorithms were applied to highlight the most important features contributing to ginger inhibition on the SKOV-3 cell proliferation. Moreover, Real-Time PCR was performed to assay p53, p21 and bcl-2 genes expression. For co-expression meta-analysis of p53, mutual ranking (MR) index and transformation to Z-values (Z distribution) were applied on available transcriptome data in NCBI GEO data repository.

***Results:*** The ginger extract significantly inhibited cancer growth in ovarian cancer cell line. The most important attribute was 60 µg/ml concentration which received weights higher than 0.50, 0.75 and 0.95 by 90%, 80% and 50% of feature selection models, respectively. The expression level of p53 was increased sharply in response to ginger treatment. Systems biology analysis and meta-analysis of deposited expression value in NCBI based on rank of correlation and Z-transformation approach unraveled the key co-expressed genes and co-expressed network of P53, as the key transcription factor induced by ginger extract. High co-expression between P53 and the other apoptosis-inducing proteins such as CASP2 and DEDD was noticeable, suggesting the molecular mechanism underpinning of ginger action.

***Conclusion:*** We found that the ginger extract has anticancer properties through p53 pathway to induce apoptosis.

## Introduction

Ovarian cancer is the main reason of death from gynaecological malignant tumors, worldwide. Although there are advanced improvements in surgical techniques and accurately designed chemotherapy regimens, reversion remains practically unavoidable in patients with progressive disease.^1,2^ Ovarian cancer is the fifth cause of death related to the cancer in women and covers a histologically and genetically a wide range of malignancies, containing those of epithelial, sex cord-stromal and germ cell source.^3^ In the year 2016, about 22,280 new cases with ovarian cancer were diagnosed and approximately 14,240 women died because of this cancer in the United States.^4^

There are different kinds of ovarian cancer depend on where the cell type originated. Epithelial cell ovarian cancer (EOC), gonadal-stromal, and germ cell make 90%, 6% and 4% incidence of ovarian cancer in patients, respectively. Epithelial ovarian cancer is derived from the celomic epithelium or mesothelium (epithelial ovarian carcinoma) and others arise from primordial germ cells, ovarian stromal or mesenchyme and sex cord.^5-7^ Some factors are associated with a high risk of ovarian cancer, such as old age, nuliparity, family history, infertility and endometriosis; on the other hand, factors such as usage of oral contraceptives, salpingo-oopherectomy, tubal ligation, hysterectomy and breast feeding are known to have a more protective effect.^5,7,8^

Due to the lack of specific symptoms, the most ovarian cancers are diagnosed in the advanced stages. Therefore, the cost of treatment is high and prognosis is poor.^5^ The majority of women whose diseases are at high risk (poorly differentiated or presence of malignant cells in as cites fluid) benefit from postoperative chemotherapy. Combination chemotherapy is recommended for these patients.^8^ Chemotherapy is useful as an adjunct to surgery in some types of ovarian cancers and may be curative. Unfortunately, some factors such as severe disability, old age, malnutrition or direct organ involvement by primary or metastatic cancer influence the incidence of severe side effects of chemotherapy; therefore, using traditional medicine with chemotherapy not only kills cancer cells but also limits the cancer side effects. Ginger is from the rhizome of Zingiber officinale that has been used in traditional medicine for a long time.^9^

Great progresses in biotechnology and molecular biology have been caused the understanding of the genetics and molecular basis of disease which can help to find strategic therapeutic approaches and novel targeted therapies to manage ovarian cancer. Therefore, it might be possible to choose medications based on the molecular characteristics of tumors and also as basis of personalized medicine. Numerous experimental studies have been conducted in the chemo preventive belongings of ginger and their mechanisms. Their main focus is on antioxidant, neuroprotection, proliferation suppression, cancer prevention, pro-apoptotic and anti-inflammatory activities.^10-16^ The result of a study on the major extracts of ginger shows that 6-gingerol inhibits angiogenesis in the human endothelial cells, it also down-regulates cyclin D1 and causes cell cycle arrest in the G1 phase.^17^ In addition, 6-gingerol plays a rule in oxidative stress, DNA damage, G2/M cell cycle arrest and also it induces autophagy and activates tumor suppressor proteins including P53 and P21.^18^ Despite the anticancer activity of ginger, its mechanisms are still poorly understood.

This study focuses on the effects of the ginger extraction on human ovarian cancer cell line (SKOV-3) to find out if the new ginger extraction is effective in treatment of ovarian cancer. In addition, bioinformatics analysis was applied on these datasets to highlight the most important features contribute to ginger inhibition on the SKOV-3 cell proliferation. The expression of p21 (cyclin-dependent kinase inhibitor 1), p53 (tumor suppressor gene), and Bcl-2 (B-cell lymphoma 2) genes following ginger treatment have been investigated. Also, Systems biology analysis and meta-analysis of deposited expression value in NCBI based on rank of correlation and Z-transformation approach were applied for further investigations about effect of ginger extract treatment on ovarian cancer cell line.

## Material and Methods

### Cell culture 

SKOV-3, human epithelial ovarian cancer cell line was purchased from Pasteur Institute Cell Bank of Iran. The cells were grown as monolayer in 25 cm^2^ flask (Orange Scientific) with culture medium (DMEM) (Sigma; Chemical Co., St. Louis, MO, USA) supplemented with 10% heat-inactivated fetal bovine serum (FBS) (Gibco- Life technologies), streptomycin (100 μg/mL), penicillin (100 units/mL) (Sigma), and cultured under standard condition at 37°C in a 5% humidified CO_2_ incubator. The medium was exchange twice a week.

### Cell proliferation assay 

The effect of ginger inhibition on the SKOV-3 cell proliferation was determined by MTT (3-(4,5-Dimethylthiazol-2-yl)-2,5-DiphenyltetrazoliumBromide) assay. The cells were seeded in 96-well tissue culture plates at a density of 3500 cells per well and incubated at 37 °C and 5% CO_2_ humidified incubator. After 50% confluency, the cells were treated with the ginger extract (Sigma-Aldrich., W252108) in different concentrations and incubated for 24, 48 and 72 hours in assorted plates. Following the appropriate times, the upper medium was removed and 0.5 mg/ml of MTT (Sigma) solution (PBS and medium) was added to each well and incubated for 4h at 37°C. The medium was removed and the blue formazan crystals were dissolved in 100μl of DMSO. The absorbance was read in a microplate reader (Biotek, model Elx808) at 570 nm. Each experiment was repeated in triplicate format, and results were expressed as means ± SEM.

### Attribute weighting

As described before the inhibitory effects of ginger extracts on the SKOV-3 cell proliferation were determined by MTT assay. MTT assay was performed as described above. The absorbance was read by a microplate reader at 570 nm. Each experiment was repeated in triplicate format. In order to identify the most important attributes and to find the possible patterns in features which determine the effect of ginger inhibition on the SKOV-3 cell proliferation by MTT, 10 different algorithms of weighting models were applied on the datasets. Dataset imported into software (RapidMiner 5.0.001, Rapid-I GmbH, Stochumer Str. 475, 44,227 Dortmund, Germany). The attribute weighting models were: weight by information gain, weight by information gain ratio, weight by rule, weight by deviation, weight by chi squared statistic, weight by Gini index, weight by uncertainty, weight by relief, weight by principal component analysis (PCA), and weight by Support Vector Machines (SVM). The algorithms definitions have already been described in our previous paper.^19^ Weights were normalized into the interval between 0 and 1 to allow the comparison between different methods.

### Decision Tree Models

Decision tree algorithms provide visual explanation of the most important features through depicting an inverted tree with the most important feature as root and other variables as leaves. Various decision trees including Random Forest, Decision Stump Decision, Iterative Dichotomiser 3 (ID3), CHi-Squared Automatic Interaction Detection (CHAID) and Random Tree were applied on dataset. Details of each decision tree model have also been presented before.^19^

### RNA extraction and c-DNA synthesis 

SKO-V cells were seeded 300000 cells per 6 well. After one day, the cells were treated with 30 μg/ml ginger extract. Forty-eight hours after treatment, the upper medium was removed from monolayer cancer cells and scrapped in 1 ml RNAX-PLUS (Cinagene, Iran). RNA was completely extracted from samples using Cinagene Kit based on the manufacturer’s instruction (RNX-Plus Solution, SinaClon, Iran). After purification and quantification, RNA was determined by measuring optical density at 260 and 280 nm by nanodrop (NanoDrop- ND-1000). The cDNA synthesis was performed according to cDNA syntheses kit instruction (Qiagene).

### Real-time PCR 

Real-time PCR was carried out to detect mRNA expression^20^ with some modifications. p53, p21 and bcl-2 mRNA expression were investigated using Cycler IQ5 Multicolor Real-time PCR Detection System (Bio-Rad, USA). For various mRNA, first-strand cDNA was amplified using P53, p21 and bcl2 primers as described in the Table 1. β-actin was used as housekeeping gene. Each experiment was repeated in triplicate format, and the results were expressed as means ±SEM.

### Statistics 

Statistical analysis was performed with SPSS version 16.0 software and ANOVA test was used to compare between groups. Data are represented as Mean ± SEM. The differences were considered significant when *P<0.05.

### Co-expression based meta-analysis and co-expression network construction

For co-expression meta-analysis of p53 (Tp53), mutual ranking (MR) index and transformation to Z-values (Z distribution) were applied on available transcriptome data in NCBI GEO, as previously described.^21^ MR index is a more reliable index in meta-analysis, compared to Pearson correlation coefficient, as it is based on rank of correlation and geometric average of the Pearson correlation coefficient rank.^22^ Geometric average is a as correlation coefficient are raked in logarithmic manner.^22^ Lower amount of MR implies higher correlation and a more strong expression association. To perform co-expression meta-analysis, the deposited transcriptome data in NCBI GEO NCBI were subjected to MR and Z-transformation using COXPRESSdb.^23^ to identify the top 100 co-expressed genes with p53 transcription factor with low MR. Calculated MR associations, as meta-analysis co-expression measurement, were used for construction of co-expression network.

**Table 1:** Primers used for Real time- PCR

**Gens**	**Genes Primer sequence (5' to 3')**
P53	Forward‏:GTTCCGAGAGCTGAATGAGG Reverse: ACTTCAGGTGGCTGGAGTGA
P21	Forward: GCTTCATGC CAG CTACTTCC Reverse: CCCTTCAAAGTG CCATCTGT
Bcl-2	Forward: GTCATGTGTGTGGAGAGCGT Reverse: ACAGTTCCACAAAGGCATCC
β-actin	Forward: CCTTCCTTCCTGGGCATG Reverse: TCCTGTCGGCAATGCCAG

## Results

### The effect of ginger on cellular proliferation

**Figure 1:**
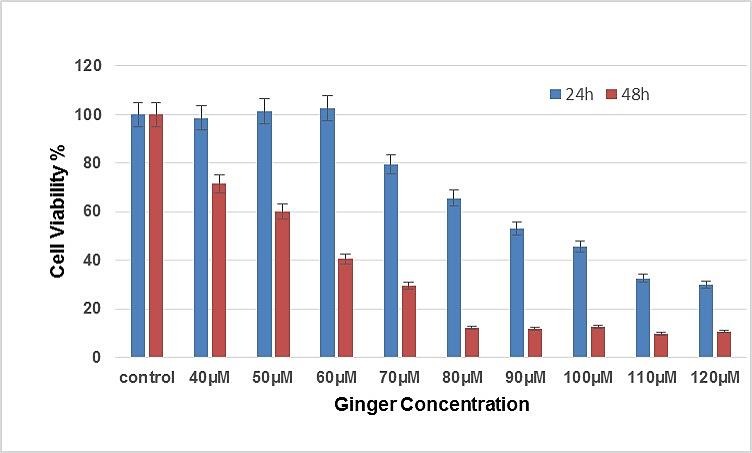
MTT assay was used to assess the effects of ginger in the Proliferation of SKOV-3 Ovarian Cancer Cell Line after 24h and 48h. There are significant differences between treated cells and controls (P<0.05)*.

**Figure 2:**
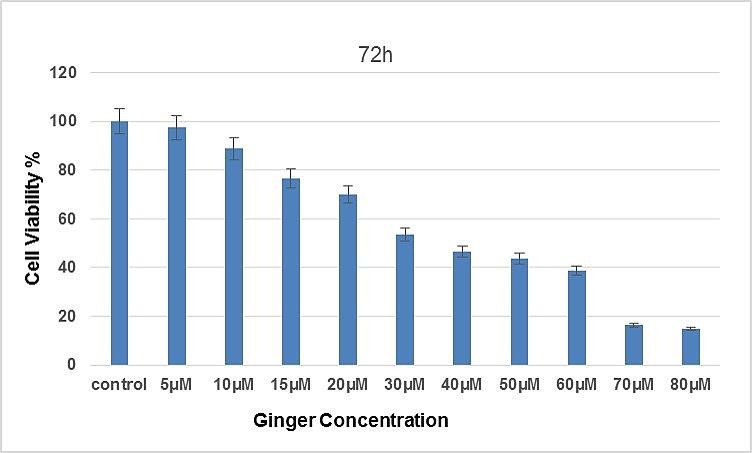
MTT assay was used to assess the effects of ginger on the Proliferation of SKOV-3 Ovarian Cancer Cell Line after 72h. There are significant differences between treated cells and controls (P<0.05)*.

In order to determine the effect of ginger on the SKOV-3 cell lines proliferation, MTT assay was illustrated at 24, 48 and 72 hours after ginger treatment. As shown in Figure 1 and 2 cell growth was inhibited considerably by ginger*;* consequently, it can be seen in figures, cell proliferation was decreased to 50% (P<0.05) after 48 and 72 hours of treatment. The results from analysis of the data for cell viability assay via MTT demonstrated that at 24h, 48h and 72h time points, the IC50 of ginger for SKOV-3 was approximately 97 µg/ml, 60 µg/ml and 40 µg/ml. respectively.

### Attribute weighting

Following normalization, 10 different attribute weighting models (as described in material and methods) were applied on GAD and RSD datasets. Each attribute was weighted between 0 and 1. These weights determined the importance of attributes in effect of new ginger extract concentration on SKOV3 cancer cell line. Attributes which gained weight equal to 0.5 or higher by at least five weighting models were selected. Table 2 shows the most important attributes was 70µg/ml concentration which received weights higher than 0.50, 0.75 and 0.95 by 90%, 80% and 50% feature selecting models. Concentration of 60µg/ml and 50µg/ml variables were the second and third important features, while 40 µg/ml concentration granted the lowest weights by attribute weighting algorithms.

**Table 2:** 10 different algorithms of weighting models applied on the datasets‏ and new generated datasets

**PCA**	**SVM**	**Relief**	**Uncertainty**	**Gini Index**	**Chi Squared**	**Deviation**	**Rule**	**Info Gain Ratio**	**Info Gain**	**Attribute**	**Count 0.50**	**Count 0.75**	**Count0.95**
.79	1.00	.26	.68	1.00	1.00	.80	1.00	1.00	1.00	70µg/ml	9	8	5
.66	.84	.23	1.00	1.00	1.00	.59	1.00	1.00	1.00	50 µg/ml	9	7	5
.86	.65	.40	.68	1.00	1.00	.82	1.00	1.00	1.00	80 µg/ml	9	7	4
1.00	.61	.30	.51	1.00	1.00	1.00	1.00	1.00	1.00	60 µg/ml	9	7	6
.66	.68	.38	1.00	1.00	1.00	.60	1.00	1.00	1.00	90 µg/ml	9	6	5
.53	.72	.39	1.00	1.00	1.00	.44	1.00	1.00	1.00	100µg/ml	8	6	5
.37	.66	.34	.76	1.00	1.00	.26	1.00	1.00	1.00	110µg/ml	7	6	4
.31	.46	.22	.76	1.00	1.00	.23	1.00	1.00	1.00	120µg/ml	6	6	4
.44	.37	.00	.37	1.00	1.00	.36	1.00	1.00	1.00	40µg/ml	5	5	4
.00	.00	1.00	.00	.00	.00	.00	1.00	.00	.00	control	2	2	2

**Figure 3:**
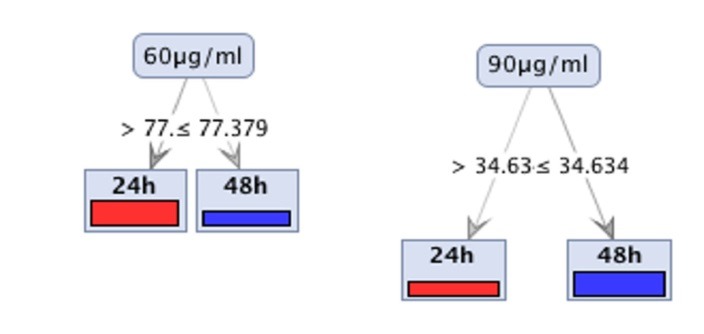
Decision Tree algorithm applied on datasets with Gini Index criterion

Tree induction algorithms also underlined the significance of features that weighed most in weighting models. Remarkably, decision tree models appointed the same features selected by attribute weighting as the root features to build the trees, as can be seen in Figure 3. The trees were just single branches showing the selected features were so decisive that can be used as cut off criteria.

**Figure 4:**
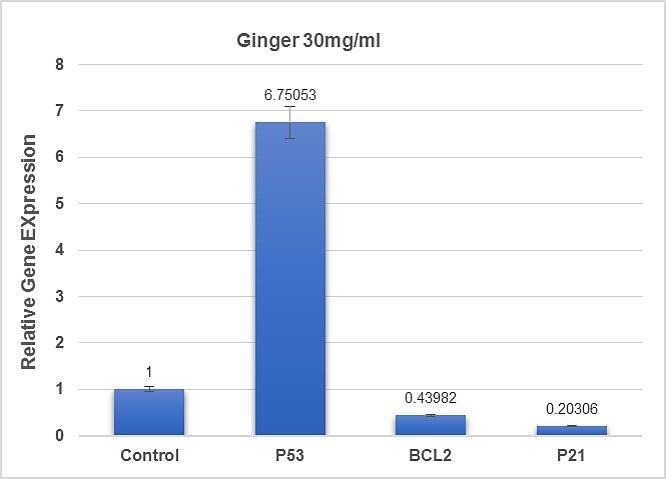
Real Time PCR Analysis: All of data were normalized to β-actin gene expression: Increase in P53 genes expression following ginger (30 μg/ml) treatment following 48h treatment.

P53, P21 and Bcl-2 genes expression in SKOV-3 cells were investigated using RT-PCR analysis (Figure 4). The genes Ct values were normalized against mRNA level of β-actin as the housekeeping gene and the relative expression for each group was measured. After 48 hours of ginger treatment, the level of p53 expression was increased.

### Co-expression based meta-analysis of p53 (Tp53) and its co-expression network 

**Figure 5:**
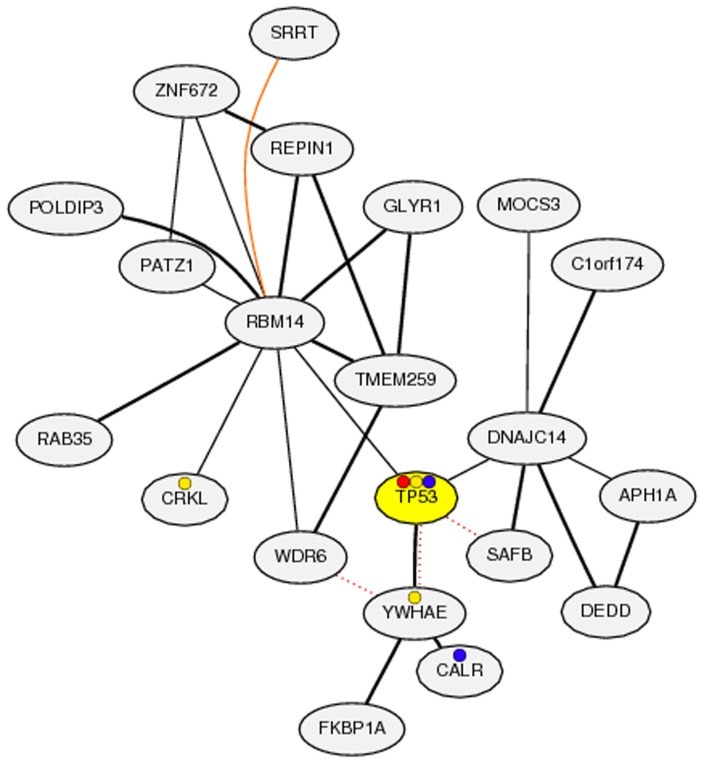
Co-expression network of Tp53, ginger associated transcription factor, derived from co-expression meta-analysis of Tp53 in transcriptomic data of NCBI GEO.

Among the studied tumor repressor genes, p53 was the top highly upregulated transcription factor in response to ginger extract, additional systems biology and meta-analysis were performed to unravel possible involved mechanism of ginger action through p53. Here, rank of correlation value was used rather than correlation value due to its reliability in meta-analysis. The top 100 co-expressed genes with p53 (Tp53) sorted based on low MR are presented in Table 3. The co-expression network, derived based on calculated association coefficients, are presented in Figure 5.

**Table 3:** The top 100 co-expressed genes with p53 (Tp53) sorted based on low mutual ranking (MR) index are presented. Meta-analysis using transcriptomic data in NCBI GEO was used for co-expression meta-analysis. When a gene list is repeatedly observed in indipendent platforms, the coexpressed gene list can be regarded as reliable with high supportability (value=3).

**Rank**	**Gene**	**Function**	**Entrez Gene ID**	**Supportability**	**MR for TP53 association**
0	TP53	tumor protein p53	7157		0
1	YWHAE	tyrosine 3-monooxygenase/tryptophan 5-monooxygenase activation protein, epsilon	7531	1	4
2	RBM14	RNA binding motif protein 14	10432	1	15.9
3	DNAJC14	DnaJ (Hsp40) homolog, subfamily C, member 14	85406	1	20.4
4	APH1A	APH1A gamma secretase subunit	51107	2	41.7
5	NONO	non-POU domain containing, octamer-binding	4841	3	42.5
6	RBBP4	retinoblastoma binding protein 4	5928	2	43.4
7	TAPBP	TAP binding protein (tapasin)	6892	3	44
8	SENP3	SUMO1/sentrin/SMT3 specific peptidase 3	26168	3	45
9	RXRB	retinoid X receptor, beta	6257	2	45.5
10	MAT2A	methionine adenosyltransferase II, alpha	4144	1	46.3
11	DEDD	death effector domain containing	9191	3	49.1
12	MAZ	MYC-associated zinc finger protein (purine-binding transcription factor)	4150	3	49.1
13	FKBP1A	FK506 binding protein 1A, 12kDa	2280	3	51
14	C21orf33	chromosome 21 open reading frame 33	8209	3	59.2
15	WDR1	WD repeat domain 1	9948	3	61.2
16	LRRC41	leucine rich repeat containing 41	10489	2	62.7
17	COLGALT1	collagen beta(1-O)galactosyltransferase 1	79709	3	64.7
18	ARHGAP1	Rho GTPase activating protein 1	392	1	72.5
19	KDELR1	KDEL (Lys-Asp-Glu-Leu) endoplasmic reticulum protein retention receptor 1	10945	3	73.1
20	CALR	calreticulin	811	2	74.2
21	GLE1	GLE1 RNA export mediator	2733	2	75.9
22	ARHGDIA	Rho GDP dissociation inhibitor (GDI) alpha	396	3	77.8
23	PATZ1	POZ (BTB) and AT hook containing zinc finger 1	23598	2	78.6
24	PRR14	proline rich 14	78994	2	80
25	RAB11B	RAB11B, member RAS oncogene family	9230	3	84.5
26	SMARCC1	SWI/SNF related, matrix associated, actin dependent regulator of chromatin, subfamily c, member 1	6599	3	84.7
27	NFYC	nuclear transcription factor Y, gamma	4802	1	85
28	FLOT2	flotillin 2	2319	3	88.6
29	STYX	serine/threonine/tyrosine interacting protein	6815	2	88.7
30	PPP5C	protein phosphatase 5, catalytic subunit	5536	2	95.2
31	TMEM259	transmembrane protein 259	91304	3	96.1
32	EIF5A	eukaryotic translation initiation factor 5A	1984	3	97.6
33	PPP2R5D	protein phosphatase 2, regulatory subunit B', delta	5528	2	98.3
34	MYBBP1A	MYB binding protein (P160) 1a	10514	3	101.4
35	PTBP1	polypyrimidine tract binding protein 1	5725	2	103
36	PHF23	PHD finger protein 23	79142	3	103.6
37	EXOSC6	exosome component 6	118460	1	104.7
38	GTF2I	general transcription factor IIi	2969	1	105.4
39	ZNF672	zinc finger protein 672	79894	2	107.1
40	TRRAP	transformation/transcription domain-associated protein	8295	3	107.3
41	CFL1	cofilin 1 (non-muscle)	1072	3	107.5
42	SAFB	scaffold attachment factor B	6294	3	107.8
43	MPDU1	mannose-P-dolichol utilization defect 1	9526	3	108.3
44	TOMM22	translocase of outer mitochondrial membrane 22 homolog (yeast)	56993	2	108.4
45	MRPL38	mitochondrial ribosomal protein L38	64978	3	109.6
46	MTMR1	myotubularin related protein 1	8776	1	112.2
47	SRSF1	serine/arginine-rich splicing factor 1	6426	3	112.6
48	PFN1	profilin 1	5216	3	114.5
49	EIF2S3	eukaryotic translation initiation factor 2, subunit 3 gamma, 52kDa	1968	3	115
50	FARSA	phenylalanyl-tRNA synthetase, alpha subunit	2193	3	116.6
51	LAMP1	lysosomal-associated membrane protein 1	3916	3	118.4
52	HNRNPH1	heterogeneous nuclear ribonucleoprotein H1 (H)	3187	3	123.3
53	STIP1	stress-induced phosphoprotein 1	10963	2	130.9
54	HSF1	heat shock transcription factor 1	3297	3	135.6
55	GANAB	glucosidase, alpha; neutral AB	23193	3	135.7
56	ASB16-AS1	ASB16 antisense RNA 1	339201	2	136
57	LIX1L	Lix1 homolog (chicken) like	128077	3	136.8
58	KLHDC3	kelch domain containing 3	116138	3	137.2
59	DRG2	developmentally regulated GTP binding protein 2	1819	3	139
60	BANF1	barrier to autointegration factor 1	8815	3	139.8
61	AKIRIN2	akirin 2	55122	1	140.8
62	RELA	v-rel avian reticuloendotheliosis viral oncogene homolog A	5970	3	141.5
63	CASP2	caspase 2, apoptosis-related cysteine peptidase	835	2	145.9
64	MAP2K2	mitogen-activated protein kinase kinase 2	5605	3	146.8
65	RANGAP1	Ran GTPase activating protein 1	5905	3	150.6
66	NAP1L4	nucleosome assembly protein 1-like 4	4676	2	151.7
67	MTA1	metastasis associated 1	9112	3	154.1
68	REPIN1	replication initiator 1	29803	2	154.3
69	ZBTB45	zinc finger and BTB domain containing 45	84878	3	155.4
70	PPP2R1A	protein phosphatase 2, regulatory subunit A, alpha	5518	3	156.1
71	CYB5R3	cytochrome b5 reductase 3	1727	2	157.6
72	UBE4B	ubiquitination factor E4B	10277	1	159.4
73	ACLY	ATP citrate lyase	47	3	160.4
74	UBE2G2	ubiquitin-conjugating enzyme E2G 2	7327	0	163.2
75	DNAAF5	dynein, axonemal, assembly factor 5	54919	3	170
76	GDI2	GDP dissociation inhibitor 2	2665	3	170.1
77	BSG	basigin (Ok blood group)	682	3	171.8
78	SLC25A11	solute carrier family 25 (mitochondrial carrier; oxoglutarate carrier), member 11	8402	3	173.4
79	BTBD2	BTB (POZ) domain containing 2	55643	3	173.7
80	C1orf174	chromosome 1 open reading frame 174	339448	2	176.2
81	ABCC1	ATP-binding cassette, sub-family C (CFTR/MRP), member 1	4363	3	178.4
82	DCAF15	DDB1 and CUL4 associated factor 15	90379	2	180.4
83	SLC29A1	solute carrier family 29 (equilibrative nucleoside transporter), member 1	2030	2	181
84	KCTD5	potassium channel tetramerization domain containing 5	54442	1	191.8
85	TBC1D5	TBC1 domain family, member 5	9779	2	192.7
86	SHC1	SHC (Src homology 2 domain containing) transforming protein 1	6464	3	192.9
87	CRTAP	cartilage associated protein	10491	2	194.3
88	NUCKS1	nuclear casein kinase and cyclin-dependent kinase substrate 1	64710	3	197.2
89	STAT2	signal transducer and activator of transcription 2, 113kDa	6773	3	198.6
90	NFRKB	nuclear factor related to kappaB binding protein	4798	2	200.8
91	ANKFY1	ankyrin repeat and FYVE domain containing 1	51479	3	207.5
92	TRAPPC1	trafficking protein particle complex 1	58485	3	208
93	CBFB	core-binding factor, beta subunit	865	2	210
94	NCOA5	nuclear receptor coactivator 5	57727	3	211.2
95	GLYR1	glyoxylate reductase 1 homolog (Arabidopsis)	84656	2	213.7
96	HNRNPU	heterogeneous nuclear ribonucleoprotein U (scaffold attachment factor A)	3192	3	213.9
97	NUCB1	nucleobindin 1	4924	3	214.7
98	NUMA1	nuclear mitotic apparatus protein 1	4926	3	216.3
99	CTNND1	catenin (cadherin-associated protein), delta 1	1500	3	216.6
100	CTNNA1	catenin (cadherin-associated protein), alpha 1, 102kDa	1495	2	217.2

YWHAE (tyrosine 3-monooxygenase) was the top co-expressed genes with P53 according to meta-analysis (Table 3, Figure 5). Interestingly, two apoptosis inducing genes, including DEDD (death effector domain containing) and CASP2 (caspase 2, apoptosis-related cysteine peptidase) are highly co-expressed with P53 which can be induced after ginger application. Based on normalized meta-data derived from expression data of different tissues and cell lines in NCBI GEO (Supplementary 1 and Supplementary 2), we calculated the Pearson correlation, in addition to MR. Highly positive and significant correlation was observed between P53 and CASP2 (Pearson correlation = 94.1%, P-Value = 0.000) and also P53 and DEDD (Pearson correlation = 90%, P-Value = 0.000).

## Discussion

In this study, we investigated the effects of the ginger extract on ovarian cancer cell line and used bioinformatics analysis to find out the most accurate and reliable results. Ginger (Zingiber officinale), a natural poly-phenol constituent from rhizomes and ginger root, is extensively used as a spice or a traditional medicine. Researchers have been consistently revealed anti-cancer activities of phenolic substance in vegetables and fruits both in vitro and in vivo.^17,24-27^ Recently, different publications reveled the anticancer effect of ginger on various human cancer cell lines such as breast cancer (BC), prostate adeno-carcinoma (PC-3), Hela (Human cervical cancer), lung non-small cancer (A549), and colon cancer.^28-32^ Weng and the colleagues reported that 6-Shogaol and 6-gingerol efficiently block invasion and metastasis of hepatocellular carcinoma by different molecular mechanisms.^26^

Our studies by MTT assay illustrated that the ginger extract displayed strong cytotoxicity effects on ovarian cancer cell line, SKOV-3. Attribute weighting algorithms weighs the importance of each attribute in distinguishing between different concentrations of ginger; the results showed a few ranges of concentrations, from 50µg/ml to 80µg/ml, gained the highest possible weights and this range can be used to find the best concentration in lab works. Decision tree models also confirmed the above findings and clearly showed that these concentrations are playing crucial roles in suppressing SKOV-3 cancer cell line toxicity.

In order to normal cells are transformed into a fully malignant cancer cells, a set of genetic and epigenetic alterations must be occurred.^33^ Genes associated with cell death program is considered crucial for the appropriate function and development of most mammalian organisms. BCL-2 (B-Cell Lymphoma 2), a member of the human Bcl-2 family is one of the main anti-apoptotic genes and seems to be a good target for cancer therapy in the future. They control the status of unreturnable for clonogenic cell survival and thereby affect tumorigenesis and host–pathogen interactions and also regulate animal development.^34-36^ Today’s clinical trials which target Bcl-2 family proteins or mRNA are giving hopes for discovering a new group of anticancer drugs.^37^ Our studies demonstrated that Bcl-2 has more than 0.4-fold reduction in expression after 48 hours ginger treatment compared to control group. Previously, Wang and colleagues in 2002 demonstrated 6-gingerol effects on apoptosis induction and inhibition of Bcl-2 expression in promyelocytic leukemia HL-60 cell.^38^

Furthermore, we investigated tumor suppressor p53 and cyclin-dependent kinase inhibitor 1 p21 genes in this study to find out their role in SKOV-3 cell death after ginger therapy. In many cell types, inactivation of the p53 gene is the most common alternation explained in ovarian cancer.^39,40^ P53 is involved in some cell pathways such as cell cycle arrest, apoptosis, metastasis, invasion, stem cell maintenance, metabolism, cell cycle and DNA repair.^41-43^ Moreover, P53-target genes play important roles in cell cycle arrest (e.g., p21) and apoptotic (e.g.; Bax) pathway.^44^ p21 is expressed by both p53-dependent and independent mechanisms after stress.^45^ In cell cycle arrest pathway, p53 affects p21 expression, thus p21 stimulation inhibits tumor development and causes cell arrest;^45,46^ however, it can be activated independently and can have cancer-promoting properties.^47^ Therefore, the control of p53's transcriptional activity is critical for novel therapeutic approaches to design drugs for ovarian cancer treatment.^47,48^

Our result showed that the level of p53 expression in the ginger extract treated ovarian cancer cell line was increased about 7-fold compared to the control group (Figure 4). On the other hand, the level of p21 expression was decreased after drug treatment., Therefore, it could be understood that p53 might regulate the cell death in other pathway. Besides, p53 regulates transcription of apoptotic target genes such as Bcl-2 and Bax.^49^ Our results revealed bcl-2 gene expression decreased in ginger treated cells, so p53 might stimulate apoptosis through bcl-2 elimination.

Additional, Systems biology analysis and meta-analysis of deposited expression value in NCBI based on rank of correlation and Z-transformation approach unraveled the key co-expressed genes and co-expressed network of P53, as the key transcription factor induced by ginger extract. High co-expression between P53 and the other apoptosis-inducing proteins such as CASP2 and DEDD was noticeable, suggesting the molecular mechanism underpinning of ginger action.

## Conclusion

Our study revealed that p53 expression is the main reason for the cytotoxicity effects of ginger in ovarian cancer cells and the cause of cell death in SKOV-3 cells. Bioinformatics analysis help to confirm and get more accurate and reliable results driven from ginger effect on the cell line and p53 expression. The data outlined the key co-expressed genes and co-expressed network of P53, as the key transcription factor induced by ginger extract.

It could be suggested that p53 in new ginger extract treated ovarian cancer cell line stimulates tumor suppression through apoptosis, rather than cell cycle arrest.

## Acknowledgments

The authors thank Faculty of Advanced Medical Science of Tabriz University for the support. This work is funded by the Women’s Reproductive Health Research Center, Al-Zahra Hospital, Tabriz University of Medical Sciences, Tabriz, Iran.

## Ethical Issues

Not applicable.

## Conflict of Interest

The authors declare no conflict of interests.
